# Effectiveness and safety of Qingfei Dayuan granules for treating influenza and upper respiratory tract infections manifested by the pulmonary heat-toxin syndrome: A multicenter, randomized, double-blind, placebo-controlled trial

**DOI:** 10.3389/fphar.2023.1133560

**Published:** 2023-03-15

**Authors:** Weinan Li, Lihan Xie, Xiaoyun Zhu, Yi Yang, Linqun Wang, Min Yang, Hengfei Li, Xucheng Li, Guangjun Yan, Xiongfei Wu, Weijun Zhao, Jilong Zhang, Gang Yang, Yufei Guo, Chengyin Li, Rui Wang, Lijun Shi, Zhili Xiong, Puming Xu, Wenwen Kong, Mengdi Cui, Xi Yang, Yuanming Ba

**Affiliations:** ^1^ College of Traditional Chinese Medicine, Hubei University of Chinese Medicine, Wuhan, China; ^2^ Hubei Provincial Hospital of Traditional Chinese Medicine, Wuhan, China; ^3^ The Affiliated Hospital of Hubei University of Chinese Medicine, Wuhan, China; ^4^ Hubei Provincial Academy of Traditional Chinese Medicine, Wuhan, China; ^5^ Department of Nephrology, The Central Hospital of Wuhan, Tongji Medical College, Huazhong University of Science and Technology, Wuhan, China; ^6^ Department of Preventive Medicine, School of Basic Medicine, Hubei University of Chinese Medicine, Wuhan, China; ^7^ Emergency Department, Wuhan Hospital of Traditional Chinese Medicine, Wuhan, China; ^8^ Jingzhou Hospital of Traditional Chinese Medicine, Jingzhou, China; ^9^ Department of Infectious Diseases, People’s Hospital of Hanchuan, Hanchuan, China; ^10^ Respiratory Department, Yichang Hospital of Traditional Chinese Medicine, Yichang, China; ^11^ Fever Clinic, Wuhan NO 1 Hospital, Wuhan, China; ^12^ Department of Respirology, Huangshi Hospital of Traditional Chinese Medicine, Huangshi, China; ^13^ Fever Outpatient Clinic, Hubei Provincial Hospital of Traditional Medicine and Western Medicine, Wuhan, China

**Keywords:** Qingfei Dayuan granules, influenza, upper respiratory tract infections, pulmonary heat-toxin syndrome, multicenter, double-blind, placebo-controlled

## Abstract

**Background:** Patients diagnosed with influenza and upper respiratory tract infections (URTIs) have similar clinical manifestations and biochemical indices and a low detection rate of viral pathogens, mixed infection with diverse respiratory viruses, and targeted antiviral treatment difficulty in the early stage. According to the treatment strategy of “homotherapy for heteropathy” in traditional Chinese medicine (TCM), different diseases with the same clinical symptoms can be treated with the same medicines. Qingfei Dayuan granules (QFDY), a type of Chinese herbal preparation included in the *TCM Diagnosis and Treatment Protocol for COVID-19 of Hubei Province* issued by the Health Commission of Hubei Province in 2021, are recommended for patients suffering from COVID-19 with symptoms of fever, cough, and fatigue, among others. Additionally, recent studies have shown that QFDY effectively alleviates fever, cough, and other clinical symptoms in patients with influenza and URTIs.

**Materials and methods:** The study was designed as a multicenter, randomized, double-blind, placebo-controlled clinical trial for treatment for influenza and URTIs manifested by pulmonary heat-toxin syndrome (PHTS) with QFDY. A total of 220 eligible patients were enrolled from eight first-class hospitals in five cities of Hubei Province in China and randomly assigned to receive either 15 g of QFDY or a placebo three times a day for 5 days. The primary outcome was the complete fever relief time. Secondary outcomes included efficacy evaluation of TCM syndromes, scores of TCM syndromes, cure rate of each single symptom, incidence of comorbidities and progression to severe conditions, combined medications, and laboratory tests. Safety evaluations mainly involved adverse events (AEs) and changes in vital signs during the study.

**Results:** Compared with the placebo group, the complete fever relief time was shorter in the QFDY group, 24 h (12.0, 48.0) in the full analysis set (FAS) and 24 h (12.0, 49.5) in the per-protocol set (PPS) (*p* ≤ 0.001). After a 3-day treatment, the clinical recovery rate (22.3% in the FAS and 21.6% in the PPS) and cure rate of cough (38.6% in the FAS and 37.9% in the PPS), a stuffy and running nose, and sneezing (60.0% in the FAS and 59.5% in the PPS) in the QFDY group were higher than those in the placebo group (*p* < 0.05). The number of patients taking antibiotics for more than 24 h in the placebo group (nine cases) was significantly higher than that in the QFDY group (one case) (*p* < 0.05). There were no significant differences between the two groups in terms of scores of TCM syndromes, incidence of comorbidities or progression to severe conditions, combined use of acetaminophen tablets or phlegm-resolving medicines, and laboratory tests (*p* > 0.05). Meanwhile, no significant difference was found in the incidence of AEs and vital signs between the two groups (*p* > 0.05).

**Conclusion:** The trial showed that QFDY was an effective and safe treatment modality for influenza and URTIs manifested by PHTS because it shortened the complete fever relief time, accelerated clinical recovery, and alleviated symptoms such as cough, a stuffy and running nose, and sneezing during the course of treatment.

**Clinical trial registration:** https://www.chictr.org.cn/showproj.aspx?proj=131702, identifier ChiCTR2100049695.

## 1 Introduction

Influenza and upper respiratory tract infections (URTIs) are both common acute respiratory tract infectious diseases with similar etiological causes and clinical symptoms (Wei et al., 2022). They are both mainly treated in accordance with associated symptoms and antiviral drugs. Studies have shown that the incidence of these two diseases, as they are easily transmissible and difficult to cure with antiviral drugs (Cao and Chen, 2019; NHC and NATCM, 2021), appears to have increased as a result of few widely approved vaccines and a low detection rate of viral pathogens (Yu et al., 2012; Han et al., 2017; Fu and Yang, 2020; Liu et al., 2022).

Traditional Chinese medicine (TCM) is characterized by the holistic view of guiding philosophy and treatment based on syndrome differentiation, which is the basic principle. It holds that the same treatment can be applied to patients with different diseases during the course of development, which is known as “homotherapy-for-heteropathy”. Over the past thousands of years of evolution, TCM has always shown remarkable therapeutic effects on a variety of acute respiratory infections (ARIs), including influenza, URTIs, severe acute respiratory syndrome (SARS) in 2003, the H1N1 pandemic in 2009, and coronavirus disease 2019 (COVID-19) in 2020 (Huang et al., 2020; Wang and Yang, 2021). Generally, TCM contributes to relieving clinical symptoms, shortening the time needed for curing and alleviating symptoms, and reducing comorbidities and the rate of progression to severe conditions (Zhao et al., 2014; Li et al., 2020; Chu et al., 2021). During the course of influenza and URTIs, numerous symptoms and signs arise, such as fever, cough, a red and sore throat, muscular soreness, headache, a stuffy and running nose, sneezing, expectoration, thirst, poor appetite, red eyes, constipation, red tongue, yellow or greasy tongue coating, and a slippery and rapid pulse (Xi et al., 2013; Cao and Chen, 2019; NHC and NATCM, 2021), which correspond to those of pulmonary heat-toxin syndrome (PHTS) in TCM in clinical practice. It should be noted that the aforementioned symptoms are broadly in line with those of COVID-19 caused by damp heat accumulating in the lungs in TCM (NHC and NATCM, 2022). As instructed by Professor Guoqiang Mei and verified by the expert group based on the theory of “homotherapy-for-heteropathy” in TCM, Qingfei Dayuan granules (QFDY), a Chinese herbal preparation, was developed right after COVID-19 hit Hubei Province and can also be used to treat influenza and URTIs incurred by PHTS.

QFDY was recorded and approved (approval no.: Z20200003) by the Hubei Provincial Medical Products Administration in 2020. In 2021, it was included in the *TCM Diagnosis and Treatment Protocol for COVID-19 of Hubei Province* as the recommended medicine for treating COVID-19 by the Health Commission of Hubei Province. An uncontrolled study (Ba et al., 2020) and a retrospective control study (Wang et al., 2020) have shown that QFDY can shorten the fever reduction time and relieve symptoms such as fatigue, cough, and pharyngeal discomfort in COVID-19 patients. Network pharmacological analysis (Zhou et al., 2020) and GO and KEGG pathway enrichment analyses demonstrated that QFDY principally regulates biological processes, such as inflammation, immune response, and apoptosis. When treating COVID-19 patients, the active components of QFDY, such as saikosaponins and glycyrrhizic acid, can enter cells infected with SARS-CoV-2; bind to transmembrane proteins, such as the S protein; and regulate various pathways, resulting in an antiviral effect.

Therefore, we designed a multicenter, randomized, double-blind, placebo-controlled clinical trial to confirm the efficacy and safety of QFDY in the treatment of influenza and URTIs manifested by PHTS.

## 2 Materials and methods

### 2.1 Investigational medications

“Type A extract” (Heinrich et al., 2022) are botanical drugs, and their extracts are included in the national or regional pharmacopoeia used as active ingredients in phytopharmaceuticals with regulated medical use (licensed, listed, or registered medicines). As a Type A extract, QFDY is composed of 13 botanical drugs and functions to clear the lungs, eliminate phlegm, and detoxify and eliminate epidemics. Through modern pharmaceutical technology, the decoctions of QFDY are then extracted, concentrated, dried, and shaped into granules. The quality control analysis of QFDY used ultra-performance liquid chromatography (UPLC) (Heinrich et al., 2020). The daily dosage of QFDY is as follows: *Bupleurum chinense* DC. [Apiaceae; Bupleuri Radix] (Rivera et al., 2014): 5.81 g (equally 20 g herb medicine, abbreviated as 20 g); *Scutellaria baicalensis* Georgi [Lamiaceae; Scutellariae radix]: 2.90 g (10 g); *Pinellia ternata* (Thunb.) Breit. [Araceae; Pinelliae Rhizoma Praeparatum]: 2.90 g (10 g); *Codonopsis pilosula* (Franch.) Nannf. [Campanulaceae; Codonopsis Radix]: 4.35 g (15 g); *Trichosanthes kirilowii* Maxim. [Cucurbitaceae; Trichosanthis Fructus]: 2.90 g (10 g); *Areca catechu* L. [Arecaceae; Arecae semen]: 2.90 g (10 g); *Lanxangia tsaoko* (Crevost & Lemarié) M.F. Newman & Škorničk. [Zingiberaceae; Tsaoko Fructus]: 4.35 g (15 g); *Magnolia officinalis* Rehder & E.H. Wilson [Magnoliaceae; Magnoliae Officinalis Cortex]: 4.35 g (15 g); *Anemarrhena asphodeloides* Bunge [Asparagaceae; Anemarrhenae Rhizoma]: 2.90 g (10 g); *Paeonia lactiflora* Pall. [Paeoniaceae; Paeoniae radix rubra]: 2.90 g (10 g); *Glycyrrhiza uralensis* Fisch. ex DC. [Fabaceae; Glycyrrhizae Radix et Rhizoma]: 2.90 g (10 g); Citrus × aurantium f. deliciosa (Ten.) M. Hiroe [Rutaceae; Citri Reticulatae Pericarpium]: 2.90 g (10 g); and *Polygonum cuspidatum* Siebold & Zucc. [Polygonaceae; Polygoni Cuspidati Rhizoma et Radix]: 2.90 g (10 g).

The trial drug QFDY and placebo (product specifications: 15 g × 15 bags) were both provided by Jing Brand Chizhengtang Pharmaceutical Co., Ltd (Huangshi City, Hubei Province, China). The placebo was composed of maltodextrin, resembling QFDY in shape.

### 2.2 Recruitment of participants

A total of 220 patients suffering from influenza and URTIs manifested by PHTS were included in this study. They were recruited from eight first-class hospitals in five cities of Hubei Province, namely, the Hubei Provincial Hospital of TCM, Hubei Provincial Hospital of Traditional Medicine and Western Medicine, Wuhan No. 1 Hospital, Wuhan Hospital of Traditional Chinese Medicine, Huangshi Hospital of Traditional Chinese Medicine, Yichang Hospital of Traditional Chinese Medicine, Jingzhou Hospital of Traditional Chinese Medicine, and People’s Hospital of Hanchuan. The recruitment work was initiated in July 2021 and terminated in December 2021.

### 2.3 Inclusion and exclusion criteria

The inclusion criteria of the study were as follows: ① met the clinical diagnostic criteria of influenza or URTIs; ② met the differentiation criteria of PHTS; ③ aged 18–70 years; ④ had a fever lasting for ≤48 h with an axillary temperature ≥37.3°C; and ⑤ informed consent process complying with the regulations, with signed informed consent. First, the clinical diagnostic criteria of influenza were formulated by referring to relevant criteria for confirmed cases of influenza in the *Diagnosis and Treatment Protocol of Influenza (2020)* issued by the National Health Commission (NHC and NATCM, 2021). Second, the clinical diagnostic criteria of URTIs in this study were designed according to the *Guidelines for Primary Care of the Acute Upper Respiratory Tract Infections (practice version 2018)* (Cao and Chen, 2019) and internal medicine (Lin et al., 2005). Clinical diagnosis can be made in accordance with the medical history, epidemiology, and symptoms and signs of the nose and throat as well as routine blood tests. Generally, there is no need for diagnosis of the etiological causes of diseases. Finally, the differentiation criteria of PHTS were formulated in line with the *Diagnosis and Treatment Protocol of Influenza (2020),* the *Guidelines for Primary Care of the Acute Upper Respiratory Tract Infections (practice version 2018)*, and the *Diagnosis and Treatment Protocol of Fever Caused by Exogenous Pathogens (upper respiratory tract infections and influenza)* (Xi et al., 2013). PHTS is a syndrome in TCM that manifests as fever, cough, a red and sore throat, muscular soreness, headache, a stuffy and running nose, sneezing, expectoration, thirst, poor appetite, red eyes, constipation, red tongue, yellow or greasy tongue coating, and slippery and rapid pulse and a series of other symptoms. In TCM, a patient with some of the aforementioned symptoms can be diagnosed with PHTS. In this study, experts from eight first-class hospitals in five cities of Hubei Province defined PHTS as having two primary symptoms, of which fever must be included, and four secondary symptoms. The primary symptoms included fever, cough, and a red and sore throat, while secondary symptoms included muscular soreness, headache, a stuffy and running nose, sneezing, expectoration, thirst, poor appetite, red eyes, and constipation.

The following exclusion criteria were used: ① severe or critical cases of influenza or diagnosed with COVID-19, pharyngoconjunctival fever, herpangina, and/or suppurative amygdalitis; ② having developed comorbidities of influenza such as sinusitis, otitis media, and/or pneumonia; ③ having taken antiviral drugs against influenza included in the *Diagnosis and Treatment Protocol of Influenza (2020)* 48 h prior to diagnosis; ④ having received systematic treatments of steroids or other immunosuppressive agents; ⑤ history of seizures or febrile convulsions; ⑥ suffering from severe malnutrition, rickets, and/or serious underlying systemic primary diseases of the heart, brain, liver, kidney, and hematopoietic system; ⑦ pregnant or lactating females with allergic constitution, allergic to multiple medicines or certain known ingredients of the medicines used for the study; and ⑧ other diseases or conditions reducing the likelihood of enrollment or complicating the process, such as failure of follow-ups due to unstable living environments.

### 2.4 Study design

**FIGURE 1:**
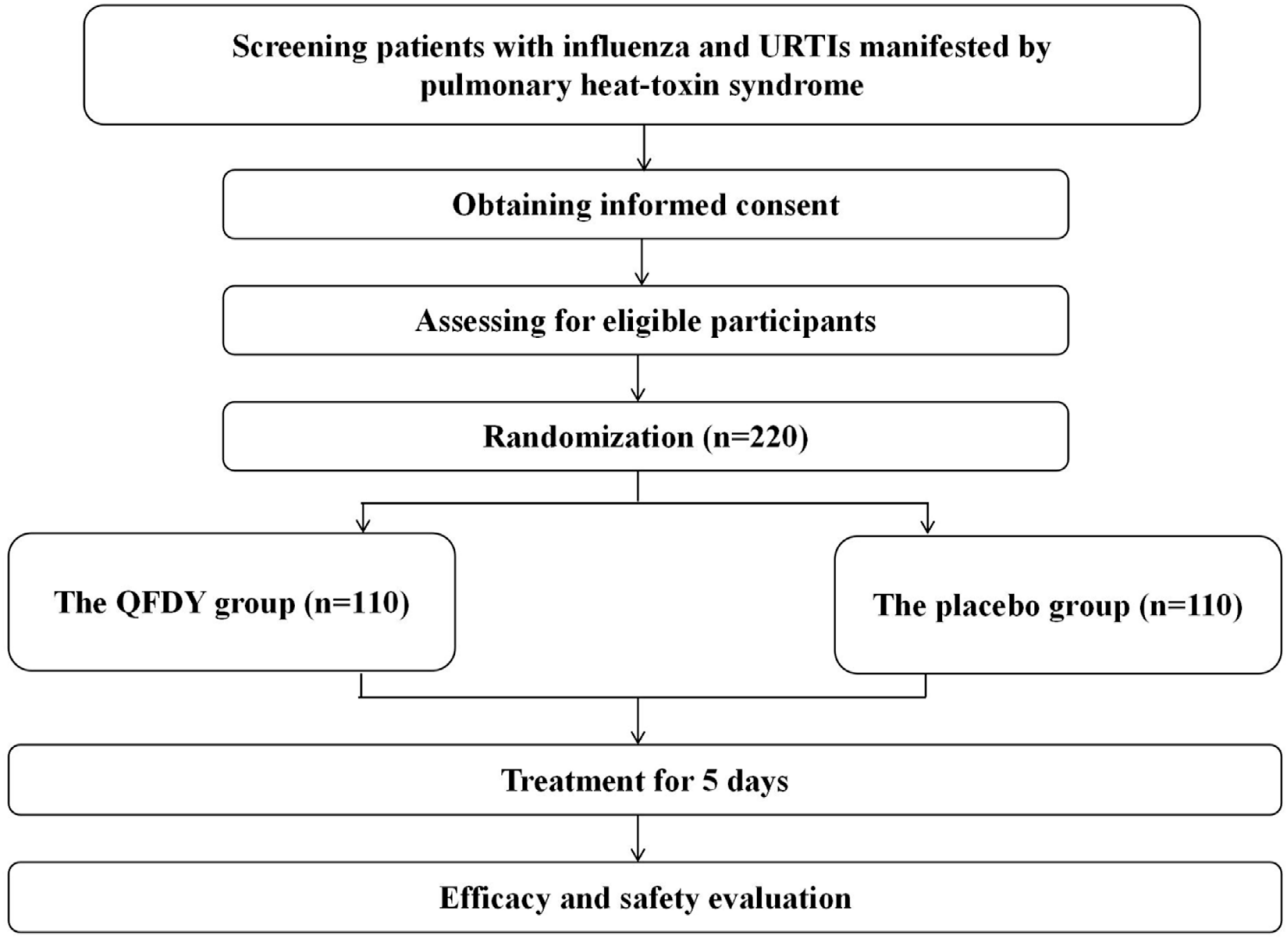
Flow diagram of the study.

Subjects were randomly assigned to two groups of the same size, in which one group received daily oral administration of QFDY (n = 110), while the other group received a placebo (n = 110). All patients received the same dosage of QFDY or placebo with warm water, 15 g, thrice a day. Patients, clinicians, investigators, and all other study personnel were blinded throughout the study. One course of treatment lasted for 5 days. Medications were discontinued earlier if fever was completely relieved and primary symptoms subsided (Figure 1; Table 1).

**TABLE 1:** Study plans of enrollment, intervention, and evaluation.

Visit project	Study period		
	Screen period (baseline)	Visit	
Time of visit (day)	−1–0	After 5 ± 1 day of medication	Follow-up of abnormal results
1. Enrollment			
Eligibility screening	×	—	—
Informed consent	×	—	—
Demographic characteristics	×	—	—
Medical examination	×	×	—
Diagnosis and medical history	×	—	—
2. Interventions			
Random allocation	×	—	—
Administration of medicines for the study	×	×	—
3. Assessment			
Clinical research log card (patients fill in it every day)	×	×	—
TCM syndrome rating table	×	×	—
Combined medication records	×	×	×
Laboratory and imaging tests	×	×	×
Adverse event monitoring**	—	×	×
4. Other work			
End of study	—	×	×

^∗∗^Throughout the study, ongoing observations and recordings of the occurrence were made.

### 2.5 Randomization and blinding

Randomization was achieved by a computer-generated random schedule in SAS software v9.4 (SAS Institute, Inc., Cary, NC, United States), with PROC PLAN performed to generate the seed. A competitive enrollment mode was adopted by subcenters. Drugs were uniformly packed and distributed in a random order. Random coding was the unique identifier given to each patient when recruited. Each drug sample was allocated with an emergency letter as a decoder. A random key in duplicate was sealed in an envelope and given to the designated administrator. At the end of the study, statistical analysts, investigators, and staff with access to the random key revealed the blindness.

### 2.6 Combined medications

Except for medicines used for the study, other medicines included in the *Diagnosis and Treatment Protocol of Influenza (2020)* and the *Guidelines for Primary Care of the Acute Upper Respiratory Tract Infections (practice version 2018)* were not allowed to be used during the observation period, including antiviral drugs, Chinese herbal medicines of the same sort or any other medicines with a potential therapeutic effect on influenza and URTIs manifested by PHTS. To protect patients with an axillary temperature above 38.5°C, investigators could provide treatments such as physical cooling and acetaminophen (≤500 mg/time; q.i.d.) instead of ibuprofen preparations according to diverse symptoms. In addition, patients could be treated with antibacterial drugs when showing indications of bacterial infection (significant increase in total leukocyte count, absolute neutrophil count, and CRP). Combined medications were set and recorded in time.

### 2.7 Outcome measures

The primary effectiveness outcome was complete fever relief time (hour). Axillary temperature was checked every 6 hours after the first administration and evaluation of treatment endpoints.

**FIGURE 2:**
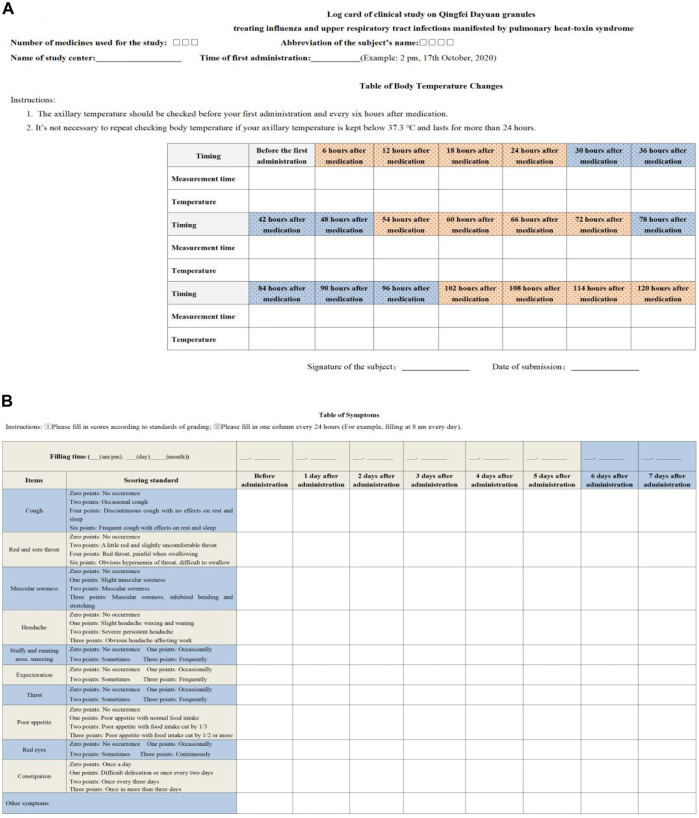
Log card of the clinical study. **(A)** is table of body temperature changes. **(B)** is table of symptoms.

Secondary effectiveness outcomes included the following (Li et al., 2021): ① therapeutic effect on TCM syndromes (effective rate), to be graded at baseline and after treatment cessation and evaluation of treatment endpoints; ② cure rate of each single symptom, time needed for curing and alleviating symptoms, to be recorded at baseline, after treatment cessation and on log cards of the clinical study, and evaluation of treatment endpoints; ③ incidence of comorbidities and progression to severe cases and evaluation of treatment endpoints; ④ negative conversion rate of the viral antigen, to be tested at baseline and after treatment cessation, and evaluation of treatment endpoints including antigen testing of influenza virus (IV-A + IV-B); antibody detection of coxsackievirus, syncytial virus, ADV, and PIV as well as serological tests of other viruses; ⑤ laboratory test results including routine blood tests, C-reactive protein, interleukin (IL), tumor necrosis factor (TNF), and lymphocyte subsets, which were examined at baseline and after treatment cessation (Figure 2).

### 2.8 Endpoint definitions and efficacy evaluation criteria

Definitions of endpoints and evaluation standards of curative effects are as follows:

(1) Complete fever relief: An axillary temperature maintained below 37.3°C after treatment that lasts for more than 24 h.
(2) Comorbidities of influenza: Pneumonia, nervous system impairment, heart injury, myositis, rhabdomyolysis, shock, otitis media, etc.
(3) Severe and critical influenza: First, a severe case of influenza can be defined as the occurrence of one of the following conditions: ① persistent hyperpyrexia lasting for more than 3 days, accompanied by terrible cough with purulent and bloody sputum or chest pain; ② quick breathing, dyspnea, and cyanosis of the lips; ③ changes in mental conditions, such as insensitivity, somnolence, restlessness, and faintness from fear; ④ serious vomiting and diarrhea with manifestations of dehydration; ⑤ complicated pneumonia; ⑥ significant aggravation of underlying conditions; or ⑦ other conditions for hospitalization.

Second, a critical case of influenza can be defined as the occurrence of one of the following conditions: ① respiratory failure; ② acute necrotizing encephalopathy; ③ shock dysfunction of various organs; or ④ other serious conditions that need monitoring treatment.

(4) Overall therapeutic effect on TCM syndromes: Evaluation criteria for cold in *the Criteria of Diagnosis and Therapeutic Effect of Disease and Syndromes in Traditional Chinese Medicine* (an industrial standard of TCM of the People’s Republic of China) (ZY/T001.1-94) were adopted here with reference to the *Guidelines for Clinical Research of New Drugs of Traditional Chinese Medicine (trial version) and the Technical Guidelines for Clinical Research of New Drugs of Traditional Chinese Medicine Treating Influenza*. The formula of the overall therapeutic effect is as follows: (score before treatment − score after treatment)/score before treatment ×100%; overall effective rate = clinical recovery rate + markedly effective rate + effective rate.

Clinical recovery: TCM symptoms and signs have totally or basically subsided, with quantitative scores of syndromes showing a decrease of at least 95%.

Marked effectiveness: Clinically, TCM symptoms and signs have been relieved significantly, with quantitative scores of syndromes showing a decrease of 70%–95%.

Effectiveness: Clinically, TCM symptoms and signs have been relieved, with the quantitative scores of syndromes showing a decrease of 30%–70%.

Ineffectiveness: Clinically, no significant improvement of TCM symptoms or signs has been found, with worsening or deteriorating conditions, with the quantitative scores of syndromes showing a decrease of less than 30%.

(5) Definition of quantitative scores of TCM syndromes: We refer to the aforementioned description of the diagnostic basis of PHTS ; the primary symptoms were classified as asymptomatic (−, zero points), mild (+, two points), moderate (++, four points), and severe (+++, six points) according to the severity of the symptoms, while the secondary symptoms were classified as asymptomatic (−, zero points), mild (+, one point), moderate (++, two points), and severe (+++, three points). The quantitative scores of TCM syndromes were the sum of all symptom scores. The research team created the quantitative rating table for PHTS in TCM (Table 2).

**TABLE 2:** Quantitative rating table for PHTS in TCM

Grading	−	+	++	+++
Primary symptoms	Zero points	Two points	Four points	Six points
Fever	Highest axillary temperature <37.3°C 24 h before diagnosis	Highest axillary temperature at 37.3°C–37.9°C 24 h before diagnosis	Highest axillary temperature at 38°C–39°C 24 h before diagnosis	Highest axillary temperature >39°C 24 h before diagnosis
Cough	No	Occasional cough	Discontinuous cough with no effects on rest and sleep	Frequent cough with effects on rest and sleep
Red and sore throat	No	Little red and slightly uncomfortable throat	Red throat; painful when swallowing	Obvious hyperemia of the throat; difficulty in swallowing

Secondary symptoms	Zero points	One point	Two points	Three points
Muscular soreness	No	Slight muscular soreness	Muscular soreness	Muscular soreness; inhibited bending and stretching
Headache	No	Slight headache waxing and waning	Severe persistent headache	Obvious headache affecting work
Stuffy and running nose; sneezing	No	Occasionally	Sometimes	Frequently
Expectoration	No	Occasionally	Sometimes	Frequently
Thirst	No	Occasionally	Sometimes	Frequently
Poor appetite	No	Poor appetite with normal food intake	Poor appetite with food intake cut by 1/3	Poor appetite with food intake cut by 1/2 or more
Red eyes	No	Occasionally	Sometimes	Continuously
Constipation	Once a day	Difficult defecation or once every 2 days	Once every 3 days	Once every 4 or more days

### 2.9 Safety evaluation

Safety evaluation mainly involved the proportion of serious adverse events (SAEs) in addition to changes in clinical significance concerning any other adverse events (AEs), vital signs, or laboratory parameters during the study. The table of AEs included in the medical record was completed honestly, including starting and ending time, duration, severity, and measures taken for outcomes. Furthermore, it was necessary to determine the correlation between AEs and medicines used for the study. Based on the severity of AEs, investigators/physicians of the study took the following measures: ① observation without discontinuation of medicines used for the study; ② observation with discontinuation of medicines used for the study without rescue therapy applied; and ③ discontinuation of medicines used for the study with rescue therapy applied. The severity of AEs was graded according to the *Common Terminology Criteria for Adverse Events* issued by the NCI of the NIH, United States Department of HHS.

### 2.10 Sample size and statistical analysis

According to relevant literature, the median time of clinical recovery following treatment with oseltamivir, a commonly used drug clinically, is 3 days (Treanor et al., 2000; Lu et al., 2021; Nong et al., 2021). The common standard deviation of the two groups was set as 36 h, and the non-inferiority margin was 12 h (*σ* = 36 h, δ = 12 h, α = 0.05, and β = 0.2). Subjects were assigned to two groups of the same size, namely, the QFDY group and the placebo group. Then, the sample size was calculated to be 105 cases/group with the non-inferiority test. It was estimated that the failure rate of follow-ups was nearly five percent, and 220 subjects were included in the study, with each group consisting of 110 patients.

In this study, a statistical software package named SPSS 23.0 was utilized to perform statistical analysis. The mean ± standard deviation/median (p25 and p75) was used to describe measurement data statistically, and frequency was used to describe count and ordinal data. For measurement data, the *t*-test and Mann‒Whitney U test were applied for comparison of groups, and the chi-square test and Fisher’s exact test were performed for comparison of count data in different groups. With regard to the statistical test method, a two-sided test was adopted with the significance level set at 0.05 (*p* = 0.05).

Three types of sets are presented in the study, namely, full analysis set (FAS), per-protocol set (PPS), and safety analysis set (SS). First, the FAS contains data from all subjects who met the inclusion criteria and were randomly divided into groups with QFDY or placebo treatment at least one time with at least one visit record. In brief, the FAS acts as an aggregation of per-protocol and drop-out cases, except for the excluded ones. Second, the PPS consists of information from subjects who meet the inclusion criteria rather than exclusion criteria and conformed to the trial protocol. Those patients with complete baseline values of primary outcomes completed all specified items in medical records, and their major variables were measured. Therefore, the PPS is the subset of the FAS. Third, the SS covers the actual data of subjects who were treated with QFDY or placebo at least once and have records of safety measures. Previous data were not allowed to be carried forward to address missing safety data. In summary, the FAS and the PPS were used to evaluate the efficacy, and the SS was used to evaluate safety.

## 3 Results

### 3.1 Baseline characteristics

As stated previously, 220 subjects from eight hospitals were included in the study. The placebo group consisted of 110 patients, with nine drop-out cases (8.18%), 101 cases completing the trial (91.82%), and no excluded cases. Among those patients in the placebo group, 101 cases were included in the FAS and 100 were included in the PPS and SS. The QFDY group was also composed of 110 patients, with seven drop-out cases (6.36%), 103 completing the trial (93.64%), and no excluded cases. The FAS covers 103 cases, and the PPS and SS cover 102 cases. Hence, no significant difference was found in the proportion of drop-out cases between the two groups (*p* > 0.05). To conclude, no significant difference was noted in the demographic characteristics at baseline (sex, age, nationality, and BMI) and clinical basic conditions (course of disease, the highest body temperature 48 h before diagnosis, the score of the highest body temperature 24 h before diagnosis, the body temperature at enrollment, virus and etiology detection, and the total score of TCM syndromes at baseline) between the two groups in both the FAS and PPS (*p* > 0.05) (Table 3).

**TABLE 3:** Descriptive statistical analysis.

Variables	Full analysis set			Per-protocol set		
	QFDY group (n = 103)	Placebo group (n = 101)	*p*-value	QFDY group (n = 102)	Placebo group (n = 100)	*p*-value
Age (year)^a^	35.0 (25.0, 54.0)	33.0 (25.0, 52.5)	0.759	34.5 (25.0, 54.0)	33.0 (25.0, 52.8)	0.802
Men^b^	43 (41.7)	54 (53.5)	0.094	42 (41.2)	54 (54.0)	0.068
Minority^b^	8 (7.8)	6 (5.9)	0.606	8 (7.8)	6 (6.0)	0.606
BMI^a^	21.48 (20.00, 23.39)	21.63 (20.08, 23.47)	0.696	21.48 (19.93, 23.37)	21.65 (20.22, 23.49)	0.570
Course of disease (hour)^a^	24 (10, 29)	24 (10, 30)	0.913	24.0 (11.5, 29.25)	24 (10, 30)	0.960
Highest body temperature 48 h before diagnosis (°C)^a^	38.0 (37.7, 38.7)	38.0 (37.7, 38.7)	0.990	38.0 (37.7, 38.7)	38.0 (37.6, 38.7)	0.987
Score of the highest body temperature 24 h before diagnosis^a^	4 (2, 4)	4 (2, 4)	0.463	3 (2, 4)	4 (2, 4)	0.462
Body temperature at enrollment (°C)^a^	37.8 (37.5, 38.5)	37.8 (37.5, 38.6)	0.563	37.8 (37.5, 38.5)	37.8 (37.5, 38.5)	0.558
Etiological testing of swab						
Influenza A virus (positive)^b^	0 (0)	0 (0)	—	0 (0)	0 (0)	—
Influenza B virus (positive)^b^	2 (1.9%)	0 (0)	0.498^c^	2 (2.0%)	0 (0)	0.498^c^
Coxsackievirus (positive)^b^	0 (0)	1 (1.0%)	0.478^c^	0 (0)	1 (1.0%)	0.478^c^
EB virus (positive)^b^	39 (37.9%)	38 (37.6%)	0.977	39 (38.2%)	38 (38.0%)	0.977
Syncytial virus (positive)^b^	2 (1.9%)	0 (0)	0.246^c^	2 (2.0%)	0 (0)	0.243^c^
ADV (positive)^b^	1 (1.0%)	0 (0)	0.497^c^	1 (1.0%)	0 (0)	0.495^c^
PIV (positive)^b^	0 (0)	2 (2.0%)	0.497^c^	0 (0)	2 (2.0%)	0.497^c^
IgM antibody to *Mycoplasma* pneumoniae (positive)^b^	3 (2.9%)	9 (8.9%)	0.082	3 (2.9%)	9 (9.0%)	0.082
Total score of TCM syndromes^a^	15 (12, 20)	17.0 (13.0, 23.0)	0.123	15.0 (12.0, 20.0)	17.0 (13.0, 22.8)	0.138

^a^ Number, mean, and standard deviation.

^b^ Number and percentage.

^c^ Fisher’s exact test.

### 3.2 Efficacy analysis

#### 3.2.1 Primary outcome measures

**FIGURE 3:**
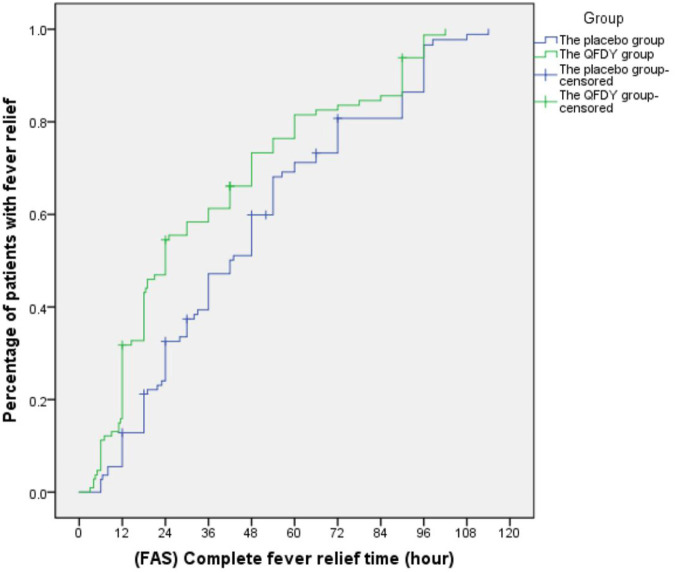
Complete fever relief time (FAS).

**FIGURE 4:**
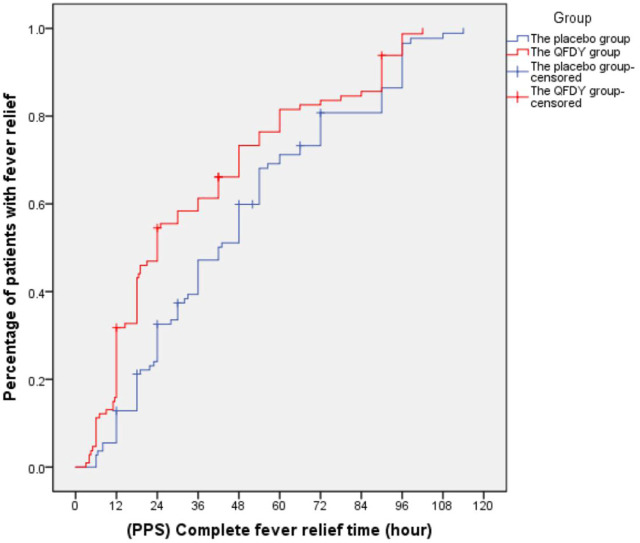
Complete fever relief time (PPS).

Primary outcomes mainly involved the complete fever relief time. In the FAS, it took the QFDY group 24 h (12.0, 48.0) to be relieved from fever completely, which was obviously shorter than the 42 h (22.4 and 66.0) it took for the placebo group. In the PPS, it took the QFDY group 24 h (12.0, 49.5) to be relieved from fever completely, which was obviously shorter than the 39 h (22.1, 66.0) it took for the placebo group. Statistically, both of the aforementioned differences were significant (*p ≤* 0.001). In conclusion, the results showed that the QFDY group was obviously superior to the placebo group in terms of complete fever relief time (Figures 3, 4).

#### 3.2.2 Secondary outcome measures

The secondary outcomes were analyzed as follows (Table 4):

① Efficacy evaluation of TCM syndromes: Differences between the two groups in both the FAS and PPS were statistically significant in terms of the clinical recovery rate (*P* < 0.05) after 3 days of treatment, but there was no significant difference in the clinical recovery rate, markedly effective rate, effective rate, and ineffective rate after 5 days of treatment between the two groups (*P* > 0.05). In summary, the results demonstrated that the QFDY group was superior to the placebo group in terms of clinical recovery rate after 3 days of treatment.
② Scores of TCM syndromes: In both the FAS and PPS, no significant difference was found between these two groups in terms of scores of TCM syndromes before and after treatment (*P* > 0.05).
③ Cure rate of each single symptom after a 3-day treatment: Differences in the two groups in both the FAS and PPS were statistically significant in the cure rate of cough, a stuffy and running nose, and sneezing (*P* < 0.05). However, no significant difference in the cure rate of a red and sore throat, muscular soreness, headache, expectoration, thirst, poor appetite, red eyes, and constipation was observed between the two groups (*P* > 0.05). In conclusion, the aforementioned results confirmed the superiority of the QFDY group to the placebo group with regard to the cure rate of symptoms such as cough, a stuffy and running nose, and sneezing after a 3-day treatment.
④ Incidence of comorbidities and progression to severe conditions: Neither complications nor severe conditions were observed in patients in either group.
⑤ Combined medications: In the two groups, no significant difference was found in the combined use of acetaminophen tablets or phlegm-resolving medicines (*P* > 0.05). The number of patients taking antibiotics for more than 24 h in the placebo group was significantly higher than that in the QFDY group (*P* < 0.05).
⑥ Laboratory tests: In the two groups, there was no significant difference in quantitative and qualitative comparisons of the negative conversion rate of viral antigen, leukocyte, C-reactive protein, interleukin-6 (IL-6), TNF, T lymphocyte, T suppressor cells, T helper cells, B lymphocyte, NK cells, and CD4+/CD8+ (*P* > 0.05).

**TABLE 4:** Analysis of secondary outcomes.

Variables	Full analysis set			Per-protocol set		
	QFDY group (n = 103)	Placebo group (n = 101)	*p*-value	QFDY group (n = 102)	Placebo group (n = 100)	*p*-value
Efficacy on TCM symptoms						
Three-day treatment						
Clinical recovery^b^	23 (22.3)	10 (9.9)	0.016	22 (21.6)	10 (10.0)	0.024
Markedly effective^b^	34 (33.0)	37 (36.6)	0.587	34 (33.3)	37 (37.0)	0.585
Effective^b^	40 (38.8)	47 (46.5)	0.266	40 (39.2)	47 (47.0)	0.264
Ineffective^b^	6 (5.8)	7 (6.9)	0.747	6 (5.9)	6 (6.0)	0.972
Five-day treatment						
Clinical recovery^b^	61 (59.2)	57 (56.4)	0.687	60 (58.8)	57 (57.0)	0.793
Markedly effective^b^	31 (30.1)	29 (28.7)	0.828	31 (30.4)	29 (29.0)	0.829
Effective^b^	10 (9.7)	11 (10.9)	0.781	10 (9.8)	11 (11.0)	0.781
Ineffective^b^	1 (1.0)	4 (4.0)	0.167	1 (1.0)	3 (3.0)	0.303
Scores of TCM syndromes						
Before treatment^a^	15.0 (12.0, 20.0)	17.0 (13.0, 23.0)	0.123	15.0 (12.0, 20.0)	17.0 (13.0, 22.8)	0.138
Five-day treatment^a^	0 (0, 3.0)	0 (0, 3.0)	0.702	0 (0, 3.0)	0 (0, 3.0)	0.846
Cure rate of each single symptom (after 3-day treatment)						
Cough^b^	34 (38.6)	19 (21.6)	0.014	33 (37.9)	19 (21.6)	0.018
Stuffy and running nose; sneezing^b^	51 (60.0)	35 (41.2)	0.014	50 (59.5)	35 (41.2)	0.017
Red and sore throat^b^	55 (56.1)	42 (46.7)	0.195	54 (70.1)	46 (57.5)	0.218
Muscular soreness^b^	55 (70.5)	46 (57.5)	0.089	54 (70.1)	46 (57.5)	0.100
Headache^b^	52 (63.4)	54 (60.7)	0.712	51 (63.0)	54 (60.7)	0.759
Expectoration^b^	38 (50.0)	28 (42.4)	0.367	37 (49.3)	28 (42.4)	0.412
Thirst^b^	44 (55.0)	41 (50.6)	0.578	43 (54.4)	41 (50.6)	0.629
Poor appetite^b^	37 (51.4)	37 (52.9)	0.861	36 (50.7)	37 (52.9)	0.798
Red eyes^b^	33 (91.7)	28 (84.8)	0.377	33 (91.7)	28 (84.8)	0.377
Constipation^b^	18 (54.5)	17 (50.0)	0.710	18 (54.5)	17 (50.0)	0.710
Incidence of comorbidities^b^	0 (0)	0 (0)	—	0 (0)	0 (0)	—
Incidence of progression to severe conditions^b^	0 (0)	0 (0)	—	0 (0)	0 (0)	—
Combined medications						
Acetaminophen tablets^b^	2 (1.9)	8 (7.9)	0.098	2 (2.0)	8 (8.0)	0.098
Within 24 h (300–500 mg)^b^	1 (1.0)	5 (5.0)	0.093	1 (1.0)	5 (5.0)	0.093
Within 24–72 h^b^	1 (1.0)	3 (3.0)	0.305	1 (1.0)	3 (3.0)	0.305
Antibiotics^b^	2 (1.9)	9 (8.9)	0.058	2 (2.0)	9 (9.0)	0.058
Within 24 h^b^	1 (1.0)	0 (0)	0.323	1 (1.0)	0 (0)	0.323
More than 24 h^b^	1 (1.0)	9 (8.9)	0.008	1 (1.0)	9 (9.0)	0.008
Phlegm-resolving medicines^b^	0 (0)	1 (1.0)	0.495^c^	0 (0)	1 (1.0)	0.495^c^

^a^ Number, mean, and standard deviation.

^b^ Number and percentage.

^c^ Fisher’s exact test.

### 3.3 Safety analysis

**FIGURE 5:**
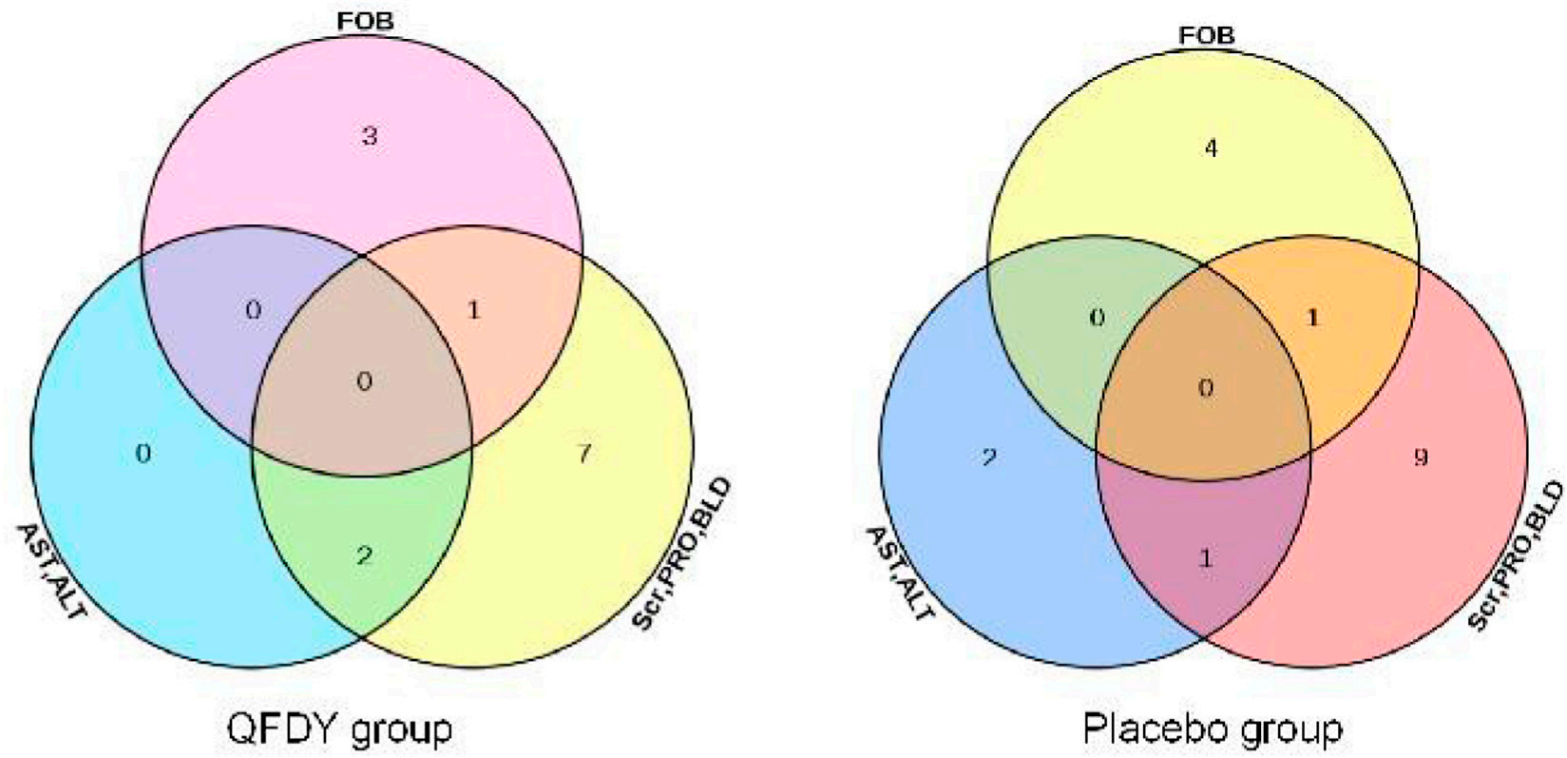
Distribution of patients with AEs.

By analyzing the SS, 30 patients were found to have mild adverse events. In the QFDY group, 16 adverse events occurred in 13 patients, including two patients with elevated serum aminotransferase levels (alanine aminotransferase, ALT or aspartate aminotransferase, AST), 10 patients with abnormal urine tests (positive urine latent blood, BLD or urine protein, PRO), and four patients with positive fecal occult blood (FOB). In the placebo group, 19 adverse events occurred in 17 patients, including three patients with elevated serum aminotransferase levels (ALT or AST), 11 patients with abnormal urine tests (positive BLD or PRO) or mild abnormal serum creatinine (Scr), and five patients with positive FOB (Figure 5).

No severe adverse events were observed during this trial. No patients in the QFDY and placebo groups had abnormalities in vital parameters (including body temperature, resting heart rate, respiration, and blood pressure) or in the laboratory tests (including myocardial enzymes, electrocardiogram, and routine blood parameters, except inflammatory markers). Therefore, it can be concluded that no significant difference was found in the incidence of AEs between the two groups (*p* > 0.05) (Table 5).

**TABLE 5:** Analysis of safety outcomes.

Adverse events	QFDY group (n = 102)	Placebo group (n = 100)
Elevated serum aminotransferase (ALT or AST)^a^	2 (2.0)	3 (3.0%)
Abnormal urine test (positive BLD or PRO) or mild abnormal Scr^a^	10 (9.8%)	11 (11.0)
Positive BLD or PRO^a^	10 (9.8)	10 (10.0)
Mild abnormal Scr^a^	0 (0)	1 (1.0)
Positive FOB^a^	4 (3.9%)	5 (5.0%)

^a^Number and percentage.

## 4 Discussion

### 4.1 Discussion of survey results

Influenza and URTIs discussed in this study fall into the common cold category in TCM. Although antiviral treatment (M2 ion channel blockers, neuraminidase inhibitors, and broad-spectrum antiviral drugs) is one of the therapies to treat such diseases, its clinical effectiveness and cost have not been fully proven by funded trials outside the field. In addition, there are still some limitations of such a treatment in controlling ARIs caused by constantly mutating viruses (Bongard et al., 2018; Butler et al., 2020; Moragas et al., 2021). Therefore, studies on TCM have been conducted to search for more natural broad-spectrum drugs for treating ARIs (Liu et al., 2022) and to verify the broad-spectrum antiviral activity of numerous plant extracts through *in vitro* experiments and tests in a mouse model. Early research showed that QFDY could effectively relieve symptoms in COVID-19 patients, including fever, cough, sore throat, muscular soreness, headache, a stuffy and running nose, sneezing, and poor appetite, which are also present in patients diagnosed with influenza and URTIs manifested by PHTS. Therefore, a multicenter, randomized, double-blind, placebo-controlled trial was designed to validate the efficacy and safety of QFDY as a broad-spectrum preparation on influenza and URTIs manifested by PHTS under the guidance of the “homotherapy-for-heteropathy” theory in TCM and a basket trial design.

The results of our trial confirmed that QFDY could shorten the time to attaining complete fever relief. After a 3-day treatment, QFDY showed obvious efficacy in terms of clinical recovery and single-symptom cure of cough, a stuffy and running nose, and sneezing. After a 5-day treatment, symptom scores in the QFDY group and the placebo group were close to 0, and laboratory tests were almost normal, making it difficult to detect differences between the two groups. Therefore, it is possible that the early use of QFDY within 3 days of diagnosis contributes to a more obvious curative effect. Meanwhile, in terms of the combined use of acetaminophen tablets or phlegm-resolving medicines, the number of patients in the QFDY group was significantly lower than that of the placebo group, but there was no statistical significance. This may prove the effect of QFDY in shortening the complete fever relief time and accelerating the recovery of symptoms, which is not related to combined medications. In addition, the number of patients taking antibiotics for more than 24 h in the placebo group was significantly higher than that in the QFDY group. This may also be associated with the effect of QFDY in shortening the complete fever relief time and accelerating the recovery of symptoms.

QFDY is composed of three TCM prescriptions, including Xiaochaihu Decoction and Xiaoxianxiong Decoction derived from Zhang Zhongjing’s *Treatise on Cold-induced Febrile Diseases* in the Han Dynasty and Dayuan Decoction from Wu Youke’s *Treatise on Warm Epidemics* in the Ming Dynasty. These decoctions are commonly used to treat common cold, fever, and respiratory infectious diseases in TCM. None of the 13 drugs in QFDY was recorded as toxic in the *Pharmacopoeia of the People’s Republic of China*. The toxicity tests of single administration and repeated administrations were both found with abnormal changes in various physiological indices of animals, indicating that the preparation was highly safe. With multiple ingredients, the Chinese herbal prescription QFDY is characterized by diverse mechanisms of action and targets in treating patients with influenza and URTIs manifested by PHTS. The crude extracts and pure compounds isolated from Bupleuri radix and Scutellariae radix exhibited various biological activities, such as anti-inflammatory, antipyretic, antibacterial, antiviral, and immunomodulatory effects (Li et al., 2011; Yang et al., 2017). Pinelliae Rhizoma Praeparatum alleviates the allergic airway inflammation of phlegm *via* regulation of the PKC/EGFR/MAPK/PI3K-AKT signaling pathway (Tao et al., 2022). Codonopsis Radix has good immune activity (Li et al., 2022). Consequently, QFDY might be a multipharmacological approach to the treatment of influenza and URTIs manifested by PHTS. However, further studies are still needed to investigate the mechanism of QFDY against influenza and URTIs manifested by PHTS.

This study aims to explore a clinical research method for influenza and URTIs manifested by PHTS based on the treatment strategy of “homotherapy for heteropathy” in TCM, which is safe and non-toxic and has great prospects in clinical application.

### 4.2 Limitations

Notwithstanding the strengths of this study, the potential limitations merit consideration. First, considering that patients with uncomplicated influenza and URTIs had a low risk of receiving the placebo treatment, most evaluation indicators were subjective clinical symptoms, the efficacy of current treatments is limited, individual differences existed, and a QFDY simulant was used as the placebo control. Additionally, the efficacy of QFDY was believed to be more reliable in this study on account of the significant superiority displayed by QFDY in contrast with its placebo, which is why no positive control groups were designed in this study. Second, the study took syndromes as the focus based on the TCM theory of “homotherapy for heteropathy” but failed to clearly define the respective proportions of patients with two different diseases during enrollment, in which QFDY was used to relieve PHTS in patients with influenza and URTIs. Third, severe and critically ill patients and those with complications were excluded from the study protocol. Fourth, the time for observation was rather short. As a result, efficacy evaluations of TCM syndromes after a 5-day treatment of one patient in the FAS and the PPS from the QFDY group and four patients in the FAS and one in the PPS were defined as invalid, so the observation of their final outcomes was not continued.

## 5 Conclusion

In summary, our findings suggest that QFDY, with favorable tolerance in the treatment of influenza and URTIs manifested by PHTS, could shorten the complete fever relief time; accelerate clinical recovery; and alleviate symptoms such as cough, a stuffy and running nose, and sneezing in the treatment process. Further investigational and experimental studies are required to reveal the specific mechanisms of QFDY and confirm its clinical efficacy.

## Data Availability

The original contributions presented in the study are included in the article; further inquiries can be directed to the corresponding author.

## Glossary

IV-A: Influenza A virus
IV-B: Influenza B virus
ADV: Adenovirus
AEs: Adverse events
ALT: Alanine aminotransferase
AST: Aspartate aminotransferase
ARIs: Acute respiratory infections
BLD: Urine latent blood
BMI: Body mass index
COVID-19: Coronavirus disease 2019
CRP: C-reactive protein
FOB: Fecal occult blood
HHS: Health and Human Services
IL: Interleukin
IL-6: Interleukin-6
NCI: National Cancer Institute
NHM: Natural herbal medicine
NIH: National Institutes of Health
NK cells: Natural killer cells
FAS: Full analysis set
PHTS: Pulmonary heat-toxin syndrome
PIV: Parainfluenza
PPS: Per-protocol set
PRO: Urine protein
QFDY: Qingfei Dayuan granules
SAEs: Serious adverse events
Scr: Serum creatinine
SS: Safety analysis set
TCM: Traditional Chinese medicine
TNF: Tumor necrosis factor
UPLC: Ultra-performance liquid chromatography
URTIs: Upper respiratory tract infections
